# Development and implementation of a novel mentoring program for clinical and translational team scientists

**DOI:** 10.1017/cts.2025.73

**Published:** 2025-04-21

**Authors:** Kathy K. Griendling, Jocelyn G. Baker, Lauren A. James, Lillian T. Eby

**Affiliations:** 1 Department of Medicine, Emory University School of Medicine, Atlanta, GA, USA; 2 Department of Psychology, University of Georgia, Athens, GA, USA; 3 Emory University School of Medicine, Atlanta, GA, USA

**Keywords:** Mentoring, team science, multidisciplinary, multi-institutional, Clinical and Translational Science Alliance

## Abstract

The importance of mentoring for professional development in Science, Technology, Engineering, Mathematics and Medicine (STEMM) fields is well established. With the increasing prevalence of team science, mentoring that incorporates team science skills is essential. Here, we describe a novel mentoring program designed to develop technical and relational/interpersonal skills for working in multi-disciplinary team science environments and to develop networks to improve collaboration in multi-disciplinary team science. The Georgia Clinical and Translational Science Alliance Translational Education and Mentoring in Science program is a nine-month program consisting of one-on-one mentoring, peer mentoring groups, mentoring resources, and shared learning experiences. Mentees (fellows) are exposed to a wide range of learning opportunities related to the goals of the program. This multi-institutional effort, in its fifth year, has been well received by participants. To date, 95 faculty, post-doctoral fellows, and research scientists have participated in the program as mentees. Participants indicated that they enjoyed the program, identified new networking opportunities, and would recommend it to others. In addition, fellows reported improved relational, technical, and networking skills over the duration of the program. Mentor ratings were also quite favorable. The multi-institutional nature of the program enriched learning and its unique structure combining traditional one-on-one mentoring with peer learning communities has been beneficial to those participating.

## Introduction

The National Academies of Science, Engineering, and Medicine 2019 consensus report highlights the importance of mentoring as a strategy for professional development in STEMM fields [1]. Similar conclusions were drawn regarding mentoring clinical and translational scientists (e.g., [2–5]). Empirical research supports this claim. In a large-scale meta-analysis of 173 primary studies and a combined 40,737 subjects, Eby and colleagues documented that mentoring is associated with many positive benefits throughout a mentee’s career, including retention, positive work attitudes, learning, and career success [6]. In addition, individuals who have been mentored report more favorable career attitudes, skill development, motivation, and performance when compared to those without experience as a mentee [7]. Moreover, the mentoring of clinical and translational scientists has gained attention within the National Institutes of Health (NIH). For example, the Clinical and Translational Science Awards Program identifies mentoring as an important approach to support the growth of a diverse clinical and translational research workforce [8].

The NIH also recognizes cross-disciplinary team science as a valuable strategy to help turn discoveries from the laboratory and clinical practice into interventions to improve individual and public health [9]. Indeed, the NIH’s National Center for Advancing Translational Science (NCATS) embraced a team science approach in their intramural and extramural funding programs. In the context of clinical and translational research, team science provides a more comprehensive perspective on health and offers insights that inform the development of health interventions [10,11].

## Program development

Based on the importance of mentoring and the recent emphasis on team science, we set out to develop a program that incorporated team science skills into a mentoring framework. In January 2018, the Collaboration and Multidisciplinary Team Science program team of the Georgia Clinical and Translational Science Alliance (Georgia CTSA) convened a committee consisting of representatives from our four institutions (Emory University, Georgia Institute of Technology, Morehouse School of Medicine and University of Georgia) to discuss currently available mentoring programs and resources and to develop an innovative program to fill identified gaps. The committee was led by a mentoring scholar (LE) and a senior faculty member with experience in developing mentoring programs for medical professionals (KG) and met monthly to consider purpose, scope, goals, target audience, reach, necessary resources, delivery mode, and desired outcomes. We paid special attention to barriers or challenges that might prevent mentors or mentees from participating, such as the substantial geographic separation of our institutions. As part of due diligence, we developed and administered a survey to prospective mentors and mentees to guide program development. The survey, which was completed by 101 potential mentors and 85 mentees, provided insight into acceptable time commitments, preferred mode of content delivery, potential program components, and barriers to participation.

## Program description

The Georgia CTSA TEAMS (Translational Education and Mentoring in Science) program was designed to provide early career faculty, post-doctoral fellows, research scientists, and clinical fellows from the CTSA member institutions the opportunity to develop professional skills in translational and clinical research, with special emphasis on skills related to working effectively in teams. This nine-month program was initially held in person, but since the 2020 pandemic, most sessions have been held virtually.

Overall objectives of the TEAMS program are to:

- Develop technical skills for working in a multi-disciplinary team science environment (e.g., grantsmanship, writing skills for interdisciplinary science)
- Develop relational/interpersonal skills for working in multi-disciplinary team science (e.g., communication skills, team management skills, cross-cultural skills)
- Develop networks to improve collaboration in multi-disciplinary team science

TEAMS has three pillars: a one-on-one mentoring match, a mentored peer learning community, and mentoring toolkits and resources. In the one-on-one mentoring arm, fellows (mentees) are matched with senior mentors based on congruence of their science, the learning objectives of the fellow, and institutional affiliation. Pairs are expected to meet monthly for the duration of the program. To facilitate relationship building, we provide planning forms for initial discussions and ongoing topics, optional mentor training on communication, information on how to be a good mentor/mentee, and curated short articles on mentoring sent throughout the duration of the program. In the learning community arm, fellows are divided into groups of 4–6 learners based on similarity in type of science and the ability to provide different perspectives to afford an opportunity for peer mentoring under the guidance of a senior advisor (the learning community mentor). Each learning community has the flexibility to create a “curriculum” that best fits its needs. The group defines topics they wish to discuss (e.g., grant writing, personal brand, time management), and the learning community mentor facilitates the discussion and identifies resources to provide relevant content. To ensure the success of the learning communities, we developed a mentor’s guide, planning forms, and a content expert database. Content experts are individuals who volunteered to speak with the learning communities on topics that fellows identify as a need. The third pillar of the TEAMS program is a mentoring toolkit that encompasses online resources and offers live training in mentoring skills. These resources are collated separately for mentors [12] and mentees [13], and include support for goal setting and alignment, being an effective mentor/mentee, communication skills, mentoring challenges, team science, diversity, relational boundaries, professional development, and mentoring best practices.

The program begins in late August with a six-hour kickoff event comprised of four sessions. First, a welcome and ice breaker introduces fellows to the program and promotes interactions and learning about others in the group. Next, we provide an overview of the learning communities, including their structure, goals, expectations, and an introduction to the mentoring toolkits. Learning community mentors join this session to meet their group and help facilitate its initial meeting. Groups are given time to introduce themselves and their learning goals, decide on a team name, set expectations for meetings and assign roles, and brainstorm their collective goals. Each group is then asked to provide a report-out so that other groups can learn from their ideas. After a short lunch break, we begin the third session, consisting of a debrief on the Strength Deployment Inventory (SDI), an on-line self-assessment tool designed to help individuals understand what motivates their behavior in relation to people, process, and performance, with the end goal of improving the quality of relationships [14]. Fellows complete the SDI in advance, and one of us (LE) is a trained facilitator for this assessment. Fellows receive their results and are educated on how to interpret them during this session. The final session is geared towards the one-on-one mentoring pillar. One-on-one mentors join us for this session, where we describe the program’s structure, goals, expectations, and resources and then provide time for the mentors and fellows to meet each other and discuss expectations for their relationship.

For the next four months of the program, we check in bi-monthly with fellows and mentors to ensure the program is functioning smoothly. Fellows are expected to meet monthly with both their one-on-one mentor and their learning community. Mentors receive emails highlighting relevant resources from the toolkit aligned with how we expect the mentoring relationship to move forward. The co-directors also hold monthly “office hours” where both learning community and one-on-one mentors can drop in and bring issues they would like to discuss.

In mid-January, we hold a second event to bring all the fellows together. This half-day symposium features a skill-building workshop (e.g., conflict management) as well as a speed mentoring session. Speed mentoring topics are chosen in advance via a survey and fellows choose the groups they want to attend. Popular topics include “How to talk to your Chair,” “Finding funding,” “Time management,” “Networking,” and “Developing leadership skills.” Each group is led by a topical expert who is asked to provide a one-page resource/tip sheet for the fellows’ use. Sessions consist of questions and answers as well as general tips to start the conversation. Each session lasts 20 minutes and then fellows rotate to their second and third choices.

As with the first half of the program, the next 4–5 months consist of monthly meetings with the mentors and the learning community. We continue to keep in touch with both groups via email and hold office hours for mentors who are having challenges. Towards the end of this period, we prepare for graduation by collecting comments from mentors and fellows describing their mentoring experience.

The TEAMS program closes with a half-day graduation event. A dynamic, inspirational, near-peer speaker is invited to address topics relevant to fellows’ career stage (e.g., “Charting Your Course as Faculty,” “Improving Work-life Balance,” “Mentorship,” and “Developing your Personal Toolkit”). Fellows are then individually recognized by their mentor with comments about the relationship, followed by individual recognition of each of the mentors and learning community mentors. Each fellow is given a certificate of completion and each mentor a small gift memorializing their contributions.

## Participant recruitment

Fellows are recruited from the Georgia CTSA partner institutions by advertising in the weekly CTSA newsletter as well as stand-alone announcements on the CTSA listserv. Outreach is also made through relevant institutional listservs. Advertising for mentors involves a similar process, but also includes individual outreach by the directors in cases where a mentor-fellow match is not obvious. Mentor applications (described below) also ask applicants to indicate their willingness to be a one-on-one mentor, a learning community mentor, and/or a content expert.

## Mentor-fellow matching

The matching process is complex due to the multi-institutional nature of the program and the wide variety of scientific disciplines represented by the fellows. During the application process, candidates provide a curriculum vitae and complete an extensive online intake form that gathers information on their knowledge of, and interest in, different possible mentoring domains, including relational skills such as conflict management, communication, managing a team, and crucial conversations, as well as technical skills like grant writing and presentation skills, and finding collaborators. We also ask for information about the candidate’s research type and area, their goals and expectations for the program, their professional strengths and what they will bring to the program. At program completion, we assess fellow knowledge on the same skills assessed at intake to measure improvement over time (via an online survey). We ask prospective mentors to complete a comparable survey that we subsequently use in matching mentor-fellow pairs. Because of the breadth of research represented by our institutions, we do not seek an exact match of scientific expertise; rather, we more heavily consider type of research (basic, preclinical, clinical, dissemination, and implementation), research focus, and in the first few cohorts we intentionally matched across institutions to increase opportunities for cross-institutional collaboration.

Learning communities are created in part based on career stage, but also with attention to diversifying the groups based on sociodemographics, research focus/type, and institution. Learning community mentors are chosen for their experience with leading small groups, their expertise in areas the fellows identified as a mentoring need, and congruence of the type of their research with that of the learning community fellows.

## Implementation

The Georgia CTSA TEAMS program launched in September 2019 with an initial cohort of 22 fellows and three learning communities from all four Georgia CTSA institutions. For the first few cohorts, we offered optional mentor training, at first during the kickoff event and later virtually during the first several weeks of the program. Feedback on mentor training was mixed; the major concern was the time commitment to attend. In the latter cohorts, we partnered with already existing local mentoring programs to offer mentor training to those who desire it (with minimal uptake). Events for the first cohort were held in-person, but we pivoted to virtual offerings for the second cohort in 2020 due to the pandemic. The introduction of the virtual option made coordination across geographically separated campuses much easier, and we have maintained this format for the mid-year and graduation events since then, returning to in-person for the kickoff in 2023 based on fellow feedback.

Administration and coordination of the program is key. In addition to the co-directors (LE and KG), a program director (LJ) manages all aspects of program implementation, including intake applications, scheduling, organizing events, mentor matching, check-in emails, virtual “drop in” sessions, and development of all program materials. The program director sends “check-in” emails every other month to fellows and mentors and occasional emails highlighting curated resources from the toolkit. She also contacts content experts for the learning communities and maintains the content expert database.

We have now graduated five cohorts, ranging from 14–22 fellows each. Learning communities are typically composed of 4–6 fellows and the mentor. The sociodemographic characteristics of the cohorts are shown in Table 1.

**Table 1.** Sociodemographic characteristics of fellows

Cohort	N	Demographics (M/F/other)	% URiM	Academic title (%)	Research Type (%)	Departments Represented (#)
1	22	68% Female/ 32% Male	41%	68% Assistant Professor 23% Post-doc/ fellow 9% Research scientist	9% B, 18% P/T, 18% C, 59% DI/PH	19
2	21	62% Female/ 38% Male	14%	67% Assistant Professor 33% Post-doc/ fellow	24% B, 24% P/T, 14% C, 52% DI/PH	12
3	16	50% Female/ 50% Male	25%	69% Assistant Professor 19% Postdoc/fellow 13% Other	50% B, 69% P/T, 13% C, 13% DI/PH	10
4	22	64% Female/ 36% Male	14%	36% Assistant Professor 55% Post-doc/fellow 9% Research scientist	36% B, 27% P/T, 50% C, 55% DI/PH	16
5	14	79% Female/ 21% Male	21%	71% Assistant Professor 14% Post-doc/fellow 14% Other	21% B, 21% P/T, 57% C, 86% DI/PH	10
All	95	64% Female/ 36% Male	23%	61% Assistant Professor 31% Post-doc/fellow 4% Research scientist 4% Other	27% B, 32% P/T, 30% C, 53% DI/ PH	38

*Note*: Percentages for Research Type do not add up to 100%, because with the exception of Cohort 1, participants could select multiple options. URiM status includes Black, Latinx, or “Other.” *B* = Basic; P/T = Preclinical/Translational; *C* = Clinical; DI/PH = Dissemination and Implementation/ Public Health.

## Evaluation and outcomes

Evaluative data are collected multiple times during the program. As described above, pre-program data are collected from the fellow and mentor intake survey, where they are asked to indicate their knowledge of specific skills that map onto the program goals. We follow up with mid-year surveys as well as a survey at program completion. This cadence allows for a pre- and post-program comparison to assess targeted skill gains and capture overall impact of, and reactions to, the program. Surveys are administered to both fellows and mentors (learning community and one-on-one) and consist of closed-ended questions answered using a Likert scale (1 = strongly disagree to 5 = strongly agree) and open-ended questions. We ask about their experience with the program, tangible and intangible outcomes, and suggestions for improvement. In addition, after each program event (kickoff, mid-year, and graduation), we ask attendees to complete a short evaluation of the event itself.

The results of the closed-ended questions are shown in Table 2. We present item-level means and standard deviations (by cohort) for fellows, one-on-one mentors, and learning community mentors. All participants rated the program highly. Fellows enjoyed participating in the program, identified new networking opportunities, thought the program should be continued, and would recommend it to others. Both one-on-one and LC mentor ratings were slightly lower, although still quite favorable. In general, neither type of mentors identified as many networking opportunities as fellows, perhaps because their participation in the program was more limited or their networks were already established.

**Table 2.** Quantitative evaluation at program completion

Question	Cohort 1 M (SD)	Cohort 2 M (SD)	Cohort 3 M (SD)	Cohort 4 M (SD)	Cohort 5 M (SD)	All M (SD)
**Fellows**	*N* = 16	*N* = 21	*N* = 12	*N* = 18	*N* = 11	*N* = 78
I enjoyed participating in the TEAMS program.	4.69 (0.48)	4.71 (0.56)	4.83 (0.39)	4.50 (0.51)	4.36 (0.67)	4.63 (0.53)
The TEAMS program should be continued.	4.88 (0.34)	4.71 (0.56)	4.92 (0.29)	4.68 (0.58)	4.50 (0.71)	4.74 (0.52)
The TEAMS program provided me with new networking opportunities.	4.44 (0.63)	4.33 (0.80)	4.58 (0.67)	4.47 (0.51)	4.00 (1.10)	4.38 (0.74)
I would recommend the TEAMS program to others.	4.81 (0.40)	4.71 (0.56)	4.75 (0.45)	4.58 (0.69)	4.45 (0.69)	4.67 (0.57)
**One-on-One Mentors**	*N* = 14	*N* = 13	*N* = 12	*N* = 15	*N* = 7	*N* = 61
I enjoyed participating in the TEAMS program.	4.43 (0.51)	4.85 (0.38)	4.50 (0.80)	4.20 (0.94)	4.57 (0.54)	4.49 (0.70)
The TEAMS program should be continued.	4.79 (0.43)	4.85 (0.38)	4.83 (0.39)	4.80 (0.41)	5.00 (0.00)	4.84 (0.37)
The TEAMS program provided me with new networking opportunities.	3.29 (0.61)	3.92 (0.95)	3.67 (1.30)	3.53 (1.46)	4.29 (0.76)	3.67 (1.11)
I would recommend the TEAMS program to others.	4.50 (0.52)	3.46 (1.13)	4.67 (0.65)	4.67 (0.49)	4.86 (0.38)	4.39 (0.84)
**LC Mentors**	*N* = 3	*N* = 3	*N* = 3	*N* = 1	*N* = 2	*N* = 12
I enjoyed participating in the TEAMS program.	4.67 (0.58)	4.33 (0.58)	4.33 (0.58)	4.00 (–)	5.00 (0.00)	4.50 (0.52)
The TEAMS program should be continued.	5.00 (0.00)	4.67 (0.58)	5.00 (0.00)	5.00 (–)	5.00 (0.00)	4.92 (0.29)
The TEAMS program provided me with new networking opportunities.	4.00 (1.00)	3.00 (0.00)	4.33 (0.58)	4.00 (–)	3.50 (0.71)	3.75 (0.75)
I would recommend the TEAMS program to others.	4.67 (0.58)	4.33 (0.58)	5.00 (0.00)	5.00 (–)	5.00 (0.00)	4.75 (0.45)

*Note*. All items measured on a 1 = strongly disagree to 5=strongly agree scale. *M* = mean; SD = standard deviation.

To uncover themes in the open-ended question on our survey, we conducted a qualitative content analysis of the suggestions for program improvement provided by one-on-one mentors and fellows. In total, 26 mentors and 49 fellows provided an answer to this question. Suggestions were first segmented into separate unique ideas, resulting in 28 suggestions from mentors and 59 suggestions from fellows. Then, using an inductive approach, one author (JB) coded each comment. These codes were reviewed by another author (LE) and disagreements were resolved via discussion. From this process, four main themes emerged from the mentors’ suggestions. These themes were related to: (1) providing additional guidance for the 1:1 relationship (9/28 comments); (2) improving the mentor/mentee fit (8/28 comments); (3) making adjustments to the program design/implementation (7/28 comments); and (4) increasing fellow engagement (4/28 comments). Suggestions provided by fellows consisted of: (1) making adjustments to the program design/implementation (26/59 comments); (2) improving the learning community component of the program (17/59 comments); (3) increasing networking opportunities for participants (7/59 comments); (4) providing additional guidance for the 1:1 relationship (5/59 comments); and improving mentor/mentee fit (4/59 comments). Selected example comments for each theme are provided in Table 3.

**Table 3.** Qualitative results: participant-reported suggestions for program improvement

	1:1 Mentors			Fellows		
Theme Title	#	%	Example Quote	#	%	Example Quote
Guidance for 1:1 Mentoring Relationship	9/28	32.14%	Maybe more guidance about what should be expected from mentors and what the mentees can expect from them (in a more formal way).	5/59	8.47%	I would suggest some more specific guidelines and structure for the individual mentor relationship. Maybe letting us know what the expectations and outcomes were for the relationship (e.g. frequency and total number of meetings)
Improve Mentor/Mentee Fit	8/28	28.57%	Better matching between mentor’s and mentee’s fields and interests	4/59	6.78%	I think my experience was not quite as good as others because while my 1-on-1 mentor was super nice and personable, we were not matched quite as well.
Program Implementation	7/28	25.00%	Maybe have a meeting of the mentors to check in along the way.	26/59	44.07%	In person meet-ups would be beneficial at some point in the program.
Fellow Engagement	4/28	14.29%	Encourage mentee to be more proactive.	–	–	–
Networking Opportunities	–	–	–	7/59	11.86%	I would have loved the opportunity to explore collaboration with other fellows more fully/formally.
Learning Communities	–	–	–	17/59	28.81%	The major area of improvement for me is around the learning communities. Dr [Name] went out of her way to secure speakers, but often times people did not show up, or did not ask questions when they were there. This limited the value of learning communities.

*Note*: # = number of times mentioned/total number of unique comments. % = percentage of unique comments related to each theme.

We then examined the mentees’ self-reported knowledge of selected team science skills targeted by the program. Table 4 shows their pre-program self-reported knowledge as well as their knowledge at the end of the program based on a three-point scale (ranging from 1 = no knowledge to 3 = extensive knowledge). The program goal relevant to each skill is indicated in the final column, mapped on its overarching program goal. A series of t-tests indicated that fellows’ knowledge of networking skills, relational/interpersonal skills, and technical skills all increased significantly over the duration of the program, with the largest gains in conflict management, creating a personal brand, and building/managing/working in a multidisciplinary team.

**Table 4.** Pre-program to post-program change in fellow skills

Skills	Pre- program Mean	Post-program Mean	Pre-Post Change	n	T value	Programing that Addresses This Skill	Relevant Program Goal
Creating your Personal Brand	1.61	2.11	0.50	66	5.42**	Learning Communities	Networks
Getting the Most out of a Mentoring Relationship	2.01	2.46	0.45	67	5.22**	Program Overall/ Resources	Networks
Finding Collaborators	1.98	2.39	0.41	66	4.74**	Program Overall	Networks
Conflict Management	1.94	2.44	0.50	68	6.06**	Kickoff and Midyear	Relational
Managing your Research Team	2.03	2.43	0.40	67	5.05**	Midyear	Relational
Communicating Expectations for a Group or Lab Team	2.10	2.45	0.35	67	4.39**	Midyear	Relational
Tips on Building/Working in/Managing an Interdisciplinary Team	1.91	2.46	0.55	68	6.41**	Midyear	Relational
Having Difficult Conversations	2.03	2.36	0.33	67	3.81**	Kickoff and Midyear	Relational
Grant Writing	2.12	2.37	0.25	67	3.40**	Midyear and Learning Communities	Technical
Identification of Granting Agencies and Program Announcements in Scientific Area	1.95	2.33	0.38	66	4.91**	Midyear	Technical
Choosing the Right Funding for your Career Stage	1.98	2.41	0.43	66	4.90**	Midyear	Technical

*Note*: Response options 1 = No Knowledge; 2 = Some Knowledge; 3 = Extensive Knowledge. Network = Networking Skills; Relational = Relational/Interpersonal Skills; Technical = Technical Skills. *p < 0.05, **p < .01.

## Resources

Annual implementation of the program is relatively inexpensive. Mentors and speakers generously donate their time. Program administration is supported by a 0.25 FTE coordinator and 0.025 FTE for each of the two faculty leads. Materials, including the SDI, in-person event costs, and tokens of appreciation for mentors, total about $5,000 per year. Mentoring resources are posted on the Georgia CTSA website and mentor and learning community guides are available to other programs interested in creating a similar mentoring program.

## Discussion and lessons learned

Overall, our five years of experience with this program indicates that we are filling a need for mentorship of scientists working in clinical and translational research and that the mentors benefit as well. The unique structure of the program combining traditional one-on-one mentoring with peer learning communities has been beneficial to those participating. Even though our institutions are spread over an 81-mile radius, cross-institutional mentoring enriched the experience of our fellows and was made manageable by the universal acceptance of virtual meetings during the pandemic.

As the program matured, small adjustments contributed to and enhanced its success, driven by survey comments (Table 3). For example, when the learning community mentors asked for additional guidance, we created a learning community mentor guide that incorporated best practices from those who served previously. We also coordinated content expert participation centrally so we could monitor learning community activity and who was serving. Another adjustment concerned mentor training. In the first year, training was mandatory, general in nature, and not specifically adapted to the nature of the program and the previous experience of the mentors. Evaluations were lukewarm. In the second year, we moved training from the kick-off event to stand-alone virtual sessions focused simply on effective communication, setting expectations, and common mentoring problems. These sessions were optional and better received. Because many mentors returned to the program year-over-year, as the program progressed and fewer mentors attended these sessions, we decided to offer training through established local programs, rather than holding sessions for few individuals, increasing the efficiency of the program.

As with many programs taking place during the pandemic, we learned that most content is easily delivered virtually, and that the convenience of virtual participation was embraced by our cohorts. At the same time, virtual programing made the networking aspects of the program more difficult, although still valuable based on year-over-year scores for networking opportunities. Some participants desired more in-person options, but by the third cohort (two years after the pandemic started), in-person attendance was severely limited, forcing us to return to virtual programing for all but the kick-off event. Holding the kick-off event in person seems to help set the stage for better interpersonal and intergroup interactions.

Another lesson learned concerned scheduling and institutional matching. When scheduling learning community meetings was left to the fellows, in some groups frustration ensued and attendance waned. Those learning communities that worked best created set meeting times and kept to their schedule, while those who scheduled meetings *ad hoc* had a harder time ensuring attendance. Although virtual meetings became a necessity and then a convenience, groups coalesced better if the first meeting, held during the kick-off, was in person. In terms of institutional matching, our initial aim was to match fellows and mentors from different partner institutions to potentially increase cross-institutional collaboration and networking. However, this approach created some unintended challenges such as feedback from some fellows that mentors from a different institution could not provide insight on university policies such as tenure and promotion. Some fellow-mentor pairs also commented that being from the same institution allowed for in-person meetings, which was desired by some fellows. Based on this feedback, in years 3–5 of the program we no longer intentionally matched across partner institutions. Instead, we ensured that the learning communities were cross-institutional and consisted of groups that had some shared interests (e.g., type/topic of research) but represented different disciplines to enhance learning.

An unexpected but welcome outcome was the fact that many mentor-fellow pairs continued their relationship beyond the program. While we appreciate and applaud these longer-term relationships, they did tend to reduce our mentor pool. In later years, we had to use our own networks and recruit mentors for some fellows.

Reviewing the outcomes of the program with respect to our program goals showed gains across all three areas: relational/interpersonal, technical, and networking. Of interest, no one component of TEAMS was responsible for these gains; rather, participants benefitted from all the events as well as their learning communities. Anecdotally, the one-on-one mentoring was probably the most popular aspect, since many pairs continued to meet beyond the limits of the program.

In conclusion, the Georgia CTSA TEAMS program has been highly successful, with strong support by the principal investigators and momentum to continue. Because of the customized nature of one-on-one and learning community mentoring received by fellows, they undoubtedly developed different skill sets by participating in the TEAMS program. Through the kickoff and mid-year events, one-on-one mentoring relationships, and learning community experiences, fellows were exposed to a wide range of learning opportunities related to effectively working on multidisciplinary teams, developing relational and interpersonal skills, and opportunities to develop networks to improve collaboration in multi-disciplinary clinical and translational team science.
